# Brush Polymer of Donor-Accepter Dyads via Adduct Formation between Lewis Base Polymer Donor and All Carbon Lewis Acid Acceptor

**DOI:** 10.3390/molecules22091564

**Published:** 2017-09-18

**Authors:** Yang Wang, Miao Hong, Travis S. Bailey, Eugene Y.-X. Chen

**Affiliations:** 1 Department of Chemistry, Colorado State University, Fort Collins, CO 80523-1872, USA; yang.wang@colostate.edu; 2 School of Fundamental Sciences, China Medical University, Shenyang 110122, China; 3 State Key Laboratory of Organometallic Chemistry, Shanghai Institute of Organic Chemistry, Chinese Academy of Sciences, Shanghai 200032, China; miaohong@sioc.ac.cn; 4 Department of Chemical and Biological Engineering, Colorado State University, Fort Collins, CO 80523-1370, USA; travis.bailey@colostate.edu

**Keywords:** Lewis acid-base adduct, poly(3-hexylthiophene) or P3HT, C_60_, donor-acceptor dyads, brush polymer, graft polymer

## Abstract

A synthetic method that taps into the facile Lewis base (LB)→Lewis acid (LA) adduct forming reaction between the semiconducting polymeric LB and all carbon LA C_60_ for the construction of covalently linked donor-acceptor dyads and brush polymer of dyads is reported. The polymeric LB is built on poly(3-hexylthiophene) (P3HT) macromers containing either an alkyl or vinyl imidazolium end group that can be readily converted into the *N*-heterocyclic carbene (NHC) LB site, while the brush polymer architecture is conveniently constructed via radical polymerization of the macromer P3HT with the vinyl imidazolium chain end. Simply mixing of such donor polymeric LB with C_60_ rapidly creates linked P3HT-C_60_ dyads and brush polymer of dyads in which C_60_ is covalently linked to the NHC junction connecting the vinyl polymer main chain and the brush P3HT side chains. Thermal behaviors, electronic absorption and emission properties of the resulting P3HT-C_60_ dyads and brush polymer of dyads have been investigated. The results show that a change of the topology of the P3HT-C_60_ dyad from linear to brush architecture enhances the crystallinity and *T*_m_ of the P3HT domain and, along with other findings, they indicate that the brush polymer architecture of donor-acceptor domains provides a promising approach to improve performances of polymer-based solar cells.

## 1. Introduction

Poly(3-hexylthiophene) (P3HT) is a widely used electron-donating material in polymer-based organic photovoltaics (OPVs), thanks to its high hole mobility (~0.1–1 cm^2^ V^−1^ s^−1^), a band gap of ~1.9 eV, and solution processability [1,2]. These OPV devices are typically based on a bulk heterojunction (BHJ) fabricated from a blend of a donor, typically a conjugated polymer such as P3HT, and an acceptor, typically a fullerene such as C_60_ and its derivatives. To improve the performance of such OPV devices, intense research has focused on optimizing the morphology of the donor/acceptor active layers, controlling the phase separation between the electron-donating and electron-accepting components, and improving the ordered assembly of the two components in the active layers. Therefore, new structural and morphological designs of P3HT-based materials have led to interesting new materials with enhanced performances in OPV devices. To break the limitation of a linear donor polymer and fulfill the diverse requirements in organic electronic devices, polymer structures have focused on the engineering of copolymers having block, graft, or branched architectures [3,4,5,6,7,8,9,10,11,12,13,14,15,16,17,18,19,20,21,22,23]. Among these structures, incorporation of the conjugated P3HT side chains into a main chain scaffold in the form of a polymer brush has been reported to increase the extent of the conjugated regions so that the resultant polymers have broadened optical absorption [24,25,26]. Therefore, brush polymers of P3HT, which can combine the benefits of the brush architecture and the good electronic properties of the linear P3HT, are interesting materials of consideration for electronic devices. There are several methods to obtain brush polymers containing the P3HT side chains, including “grafting to” methods to graft P3HT to silicon surface and “click reaction” to link P3HT to a polymer main chain [15,16,17,18,19]; “grafting from” method to graft the P3HT chains on poly(4-bromostyrene) and poly(4-vinylpyridine)-block-poly(4-iodo-styrene) by Kumada catalyst-transfer polycondensation [20,21]; and “grafting through” method, also known as the macromer method, to synthesize brush polymers of P3HT via ring-opening metathesis polymerization of the macromers containing the P3HT moiety [22,23].

Several strategies have been employed to control the morphology of the donor P3HT/acceptor C_60_ composite and thus optimize the device performance, such as thermal annealing [27], solvent treatment [28,29], blending [30,31], and inclusion of chemical reaction [32,33,34,35,36,37,38,39,40,41] to bond them together. There are several chemical reactions that have been explored to covalently link C_60_ to P3HT, for example, Vilsmeier-Haack reaction [32,33], Grignard-coupling reaction [34], Bingel reaction [35], lithiated reaction [36], and copolymerization with C_60_-containing monomer [37,38]. In this context, the facile reaction between a bulky *N*-heterocyclic carbene (NHC) and C_60_ that leads to the instantaneous formation of a thermally stable zwitterionic Lewis base (LB)→Lewis acid (LA) adduct connected via a C-C single bond [39,40] attracted our attention for possible applications of this small molecule adduct forming reaction in the synthesis of novel macromolecular electronic materials containing C_60_ or both P3HT and C_60_, due to its fast reaction rate, quantitative product (adduct) formation, and simplicity of the approach. In fact, using this facile adduct forming reaction we have recently synthesized a polymeric Lewis adduct, poly(NHC-C_60_), which was subsequently blended with P3HT to construct a donor/acceptor BHJ. The morphology of the BHJ using P3HT/poly(NHC–C_60_) is more controlled and the C_60_ domain size is much smaller and more uniformed, as compared to the BHJ directly using C_60_ with the same P3HT/C_60_ ratio [41]. Encouraged by these results, we hypothesized that the zwitterionic polymeric LB→LA adduct forming reaction could be used to build a novel brush polymer architecture having the densely grafted P3HT side chains that are also covalently bonded to C_60_ moieties, namely a homogeneous polymeric junction. Accordingly, this contribution reports the design and synthesis of a novel P3HT macromer with the vinyl-imidazolium (IM) chain end, P3HT-(CH_2_)_3_-VIM, which carries a P3HT chain, a polymerizable vinyl moiety, and an NHC precursory imidazolium function. Successful polymerization of P3HT-(CH_2_)_3_-VIM forms the corresponding brush polymer P[P3HT-(CH_2_)_3_-VIM]. Moreover, subsequent deprotonation of the IM generates the NHC LB, which reacts with the LA C_60_ via LB→LA adduct formation to build the novel brush polymer architecture, P[P3HT-(CH_2_)_3_-VIM-C_60_], consisting of the densely grafted P3HT side chains that are bonded to C_60_. Linked P3HT-C_60_ dyads as a control system that simulates the brush side chains have been also synthesized using the similar strategy, starting from the P3HT macromer with the methyl-IM end, P3HT-(CH_2_)_3_-MIM, or P3HT-(CH_2_)_3_-VIM, followed by deprotonation and adduct formation to generate C_60_-end-caped P3HT dyads, P3HT-(CH_2_)_3_-MIM-C_60_, or P3HT-(CH_2_)_3_-VIM-C_60_. Thermal behaviors and optical properties of the resulting polymeric P3HT-C_60_ dyads and brush polymer of linked dyads have also been investigated.

## 2. Results and Discussion

### 2.1. Synthesis of Macromers P3HT-(CH_2_)_3_-MIM and P3HT-(CH_2_)_3_-VIM

**Scheme 1:**
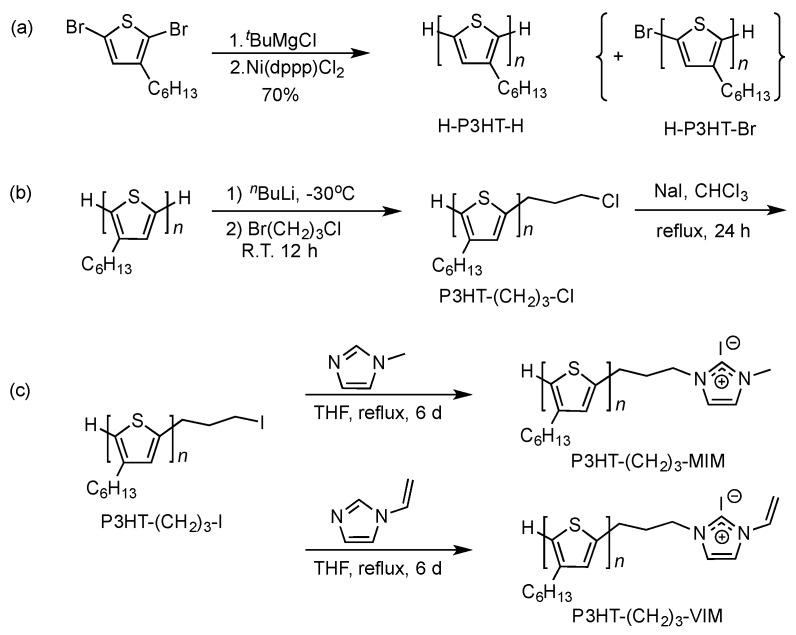
Synthetic routes to H-P3HT-H (**a**), P3HT-(CH_2_)_3_Cl (**b**), as well as macromers P3HT-(CH_2_)_3_-MIM and P3HT-(CH_2_)_3_-VIM (**c**).

**Figure 1:**
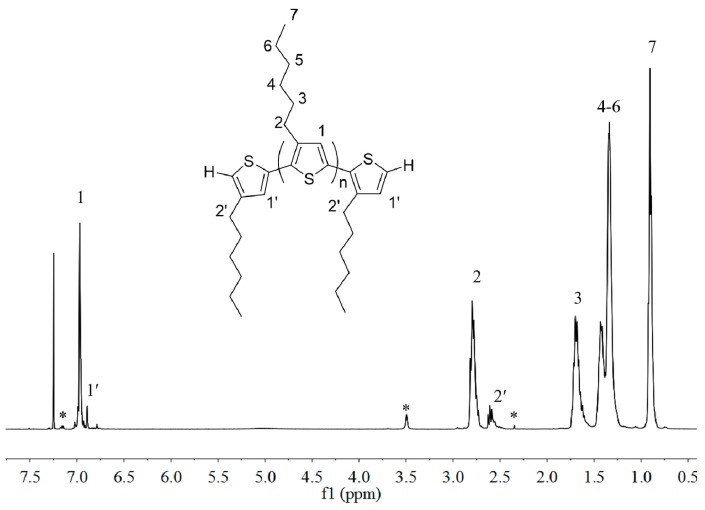
^1^H-NMR (400 MHz, CDCl_3_) spectrum of H-P3HT-H with MW = ~2000 g/mol. Peaks marked * correspond to residual solvent signals.

Macromers with two different end-groups, P3HT-(CH_2_)_3_-MIM and P3HT-(CH_2_)_3_-VIM, were synthesized following the routes outlined in Scheme 1, starting from the P3HT with H/H end ends, H-P3HT-H. This starting oligomeric material was prepared using the known quasi-living Grignard metathesis polymerization of 2,5-dibromo-3-hexylthiophene, which was first treated with *t*-BuMgCl, followed by addition of initiator Ni(dppp)Cl_2_ [42], Scheme 1a. The formation of H-P3HT-H was verified by ^1^H-NMR (Figure 1), from which the molecular weight (MW) was calculated to be ~2000 g/mol for the sample prepared by using a [monomer]/[Ni] ratio of 20/1. As usual for this method, the presence of a small amount of the P3HT with H/Br chain ends is typically unavoidable.

**Figure 2:**
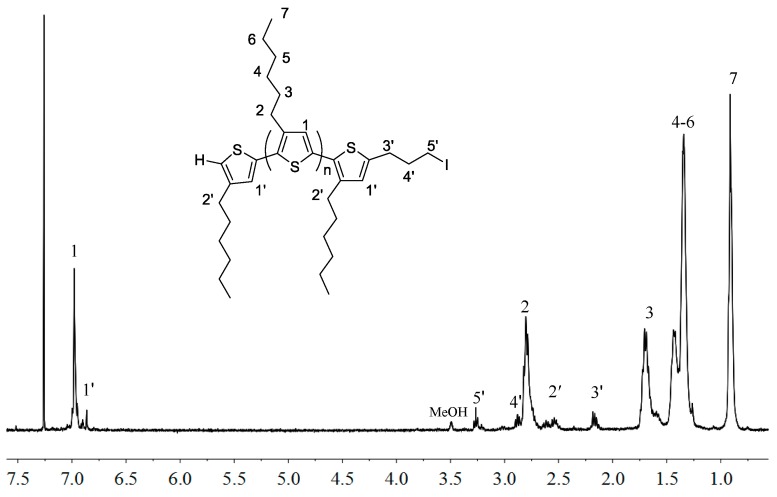
^1^H-NMR (400 MHz, CDCl_3_) spectrum of P3HT-(CH_2_)_3_I with MW = ~2000 g/mol.

**Figure 3:**
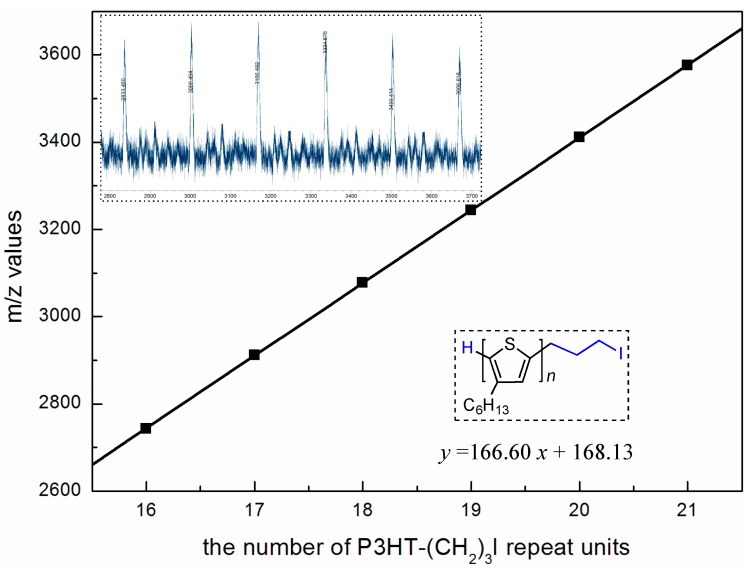
MALDI-TOF spectrum (inset) of macromer P3HT-(CH_2_)_3_I (MW~2000 g/mol by NMR) and plot of *m*/*z* values (*y* axis) vs. the number of P3HT repeat units (*x* axis).

**Figure 4:**
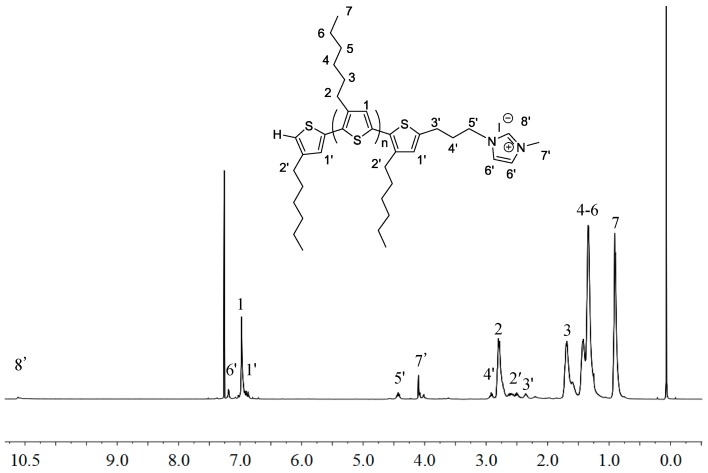
^1^H-NMR (400 MHz, CDCl_3_) spectrum of P3HT-(CH_2_)_3_-MIM with MW~2000 g/mol.

Next, the IM end-functionalization reaction was performed utilizing the active hydrogen end group on the thiophene ring. First, the lithiation reaction of H-P3HT-H with *n*-BuLi in THF at −30 °C, followed by addition of 1-bromo-3-chloropropane, led to the chloride end-functionalized P3HT, P3HT-(CH_2_)_3_Cl, in 95% isolated yield (Scheme 1b). However, this chloride intermediate is not sufficiently reactive to perform the subsequent alkylation of 1-methylimidazole or 1-vinylimidazole. To overcome this difficulty, the chloride was converted into the more reactive iodide end-functionalized P3HT-(CH_2_)_3_I in 80.8% isolated yield via halogen exchange reaction with NaI [43,44]. The appearance of new sets of the peaks at δ 3.27 (5′), 2.88 (4′) and 2.17 (3′) ppm pertaining to the end group of P3HT-(CH_2_)_3_I (Figure 2), as well as the analysis of its MALDI-TOF MS spectrum (Figure 3) confirmed the successful synthesis of P3HT-(CH_2_)_3_I. A plot of *m*/*z* values vs. the number of P3HT repeat units yielded a straight line with a slope of 166.60 and an intercept of 168.13. Thus, the slope corresponds to the mass of the P3HT monomer, whereas the intercept is the sum of the masses for the chain-end groups, H/(CH_2_)_3_I. The last step of the macromer synthesis followed the literature procedures [45], involving the nucleophilic substitution reaction of P3HT-(CH_2_)_3_I with 1-methylimidazole or 1-vinylimidazole (i.e., alkylation of imidazole) in refluxing THF for 6 days to produce macromers P3HT-(CH_2_)_3_-MIM or P3HT-(CH_2_)_3_-VIM in 84.3% or 87.6% isolated yield, (Scheme 1c). Figure 4 depicts the spectrum of P3HT-(CH_2_)_3_-MIM, clearly showing the resonances for the end-group protons of -C*H*_2_-C*H*_2_-C*H*_2_- at δ 4.43, 2.96, 2.34 ppm (labeled as protons 5′, 4′ and 3′) and for the MIM group at δ 7.19 (-C*H*=C*H*- on MIM ring) and 4.10 ppm (methyl on MIM) (labeled as protons 6′ and 7′, respectively). The resonance at δ 10.6 ppm can be readily assigned to the acidic proton of the IM ring.

**Figure 5:**
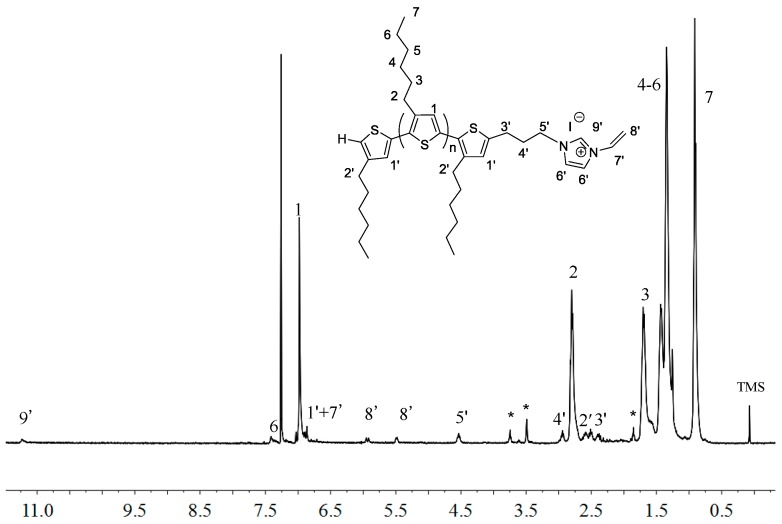
^1^H-NMR (400 MHz, CDCl_3_) spectrum of P3HT-(CH_2_)_3_-VIM with MW~2000 g/mol. Peaks marked * correspond to residual solvent signals.

Likewise, Figure 5 shows that the end group (-C*H*_2_-C*H*_2_-C*H*_2_-) of macromer P3HT-(CH_2_)_3_-VIM at δ 4.54, 2.94, 2.38 ppm (labeled as protons 5′, 4′ and3′) and the VIM group at δ 7.42 (-*CH=CH*- on IM ring, labeled as proton 6′) and δ 6.86, 5.92 and 5.48 ppm (vinyl group on IM, labeled as protons 7′ and 8′, respectively). The resonance at δ 11.2 ppm can be readily assigned to the acidic proton of the IM ring. The calculated MW by ^1^H-NMR was ~2000 g/mol. The MW measured by gel-permeation chromatograph (GPC) for the macromer P3HT-(CH_2_)_3_-VIM was somewhat higher, with *M*_n_ = 3500 g·mol^−1^ and *Đ* = 1.19.

### 2.2. Polymerization of Macromer P3HT-(CH_2_)_3_-VIM

**Figure 6:**
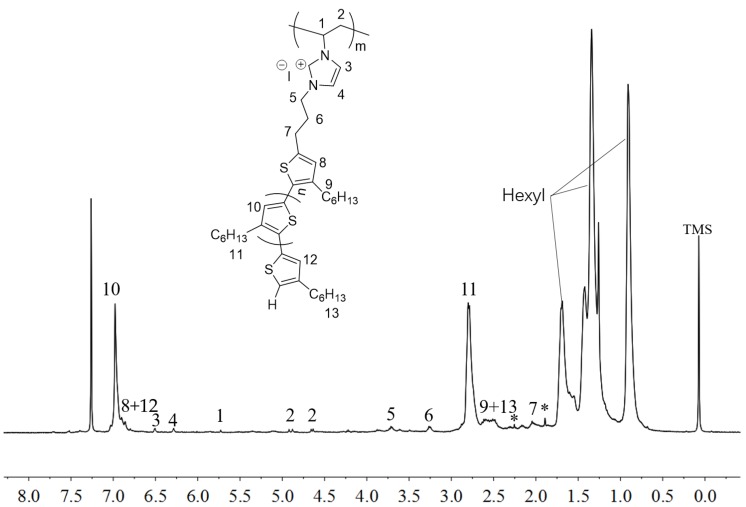
^1^H-NMR (400 MHz, CDCl_3_) spectrum of brush polymer P[P3HT-(CH_2_)_3_-VIM]. Peaks marked * correspond to residual NMR solvent signals.

**Scheme 2:**
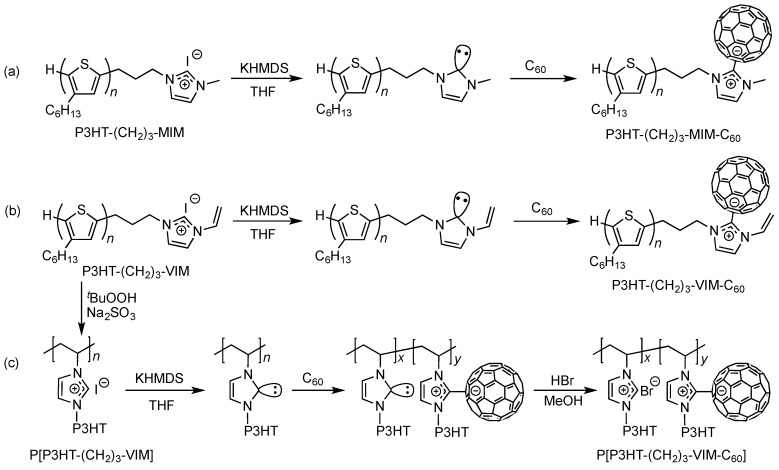
Synthetic routes to linked donor-acceptor dyads P3HT-(CH_2_)_3_-MIM-C_60_ (**a**) and P3HT-(CH_2_)_3_-VIM-C_60_ (**b**) as well as brush polymer P[P3HT-(CH_2_)_3_-VIM-C_60_] (**c**).

The polymerizability of P3HT-(CH_2_)_3_-VIM was examined by radical polymerization methods. Common radical initiators 2,2′-azobisisobutyronitrile (AIBN) and dibenzoyl peroxide (BPO) were initially employed for the polymerization study. However, AIBN failed to initiate the polymerization of macromer P3HT-(CH_2_)_3_-VIM at 80 °C in THF, and BPO did initiate the polymerization but achieved only 50% conversion of the macromer which was difficult to remove from the final polymer product due to similar solubility; this observation can be attributed to the relatively high MW of the macromer. Next, redox radical and photoinduced radical polymerization methods that possess higher activity and can initiate the polymerization under lower temperature were applied for the polymerization. To this end, di-*tert*-butyl peroxide/Na_2_SO_3_, *tert*-butyl hydroperoxide/Na_2_SO_3_, and photoinitiator 2,2-dimethoxy-2-phenylacetophenone DMPA) were tested as initiators. While di-*tert*-butyl peroxide/Na_2_SO_3_ and DMPA exhibited no polymerization activity towards the macromer, *tert*-butyl hydroperoxide/Na_2_SO_3_ not only brought about the successful polymerization but also achieved quantitative conversion of the macromere to the corresponding brush polymer P[P3HT-(CH_2_)_3_-VIM] (Scheme 2, steps (b) to (c)). Figure 6 depicts the ^1^H-NMR spectrum of P[P3HT-(CH_2_)_3_-VIM], showing that all characteristic peaks of the double bond on the VIM end group of the macromer at δ 5.92, 5.48 and 6.86 ppm disappeared, while the new peaks for the formed main chain -CH(N)CH_2_- at δ 5.73, 4.92 and 4.63 ppm (which are consistent with the reported values for a similar structure motif [46]), appeared concurrently after the polymerization. Using the GPC-MALS system, the absolute MW was measured: *M*_w_ = 3.56 × 10^4^ g·mol^−1^, *M*_n_ = 3.01 × 10^4^ g·mol^−1^, *Đ* = 1.18. These results showed that the radical polymerization of the macromer proceeded successfully to form the corresponding brush polymer P[P3HT-(CH_2_)_3_-VIM].

**Figure 7:**
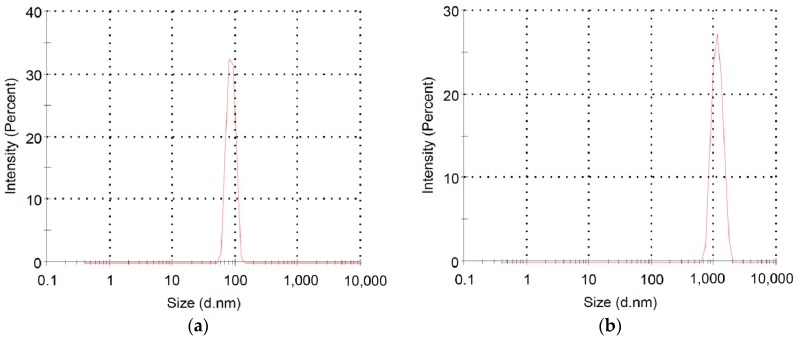
Size distributions of macromer P3HT-(CH_2_)_3_-VIM (**a**) and brush polymer P[P3HT-(CH_2_)_3_-VIM] (**b**) in CHCl_3_/MeOH (*v*/*v* = 1/2) measured by DLS at RT.

We also employed the dynamic light scattering (DLS) method to measure the change of particle sizes in solution before and after the polymerization. Figure 7 compares the particle size distribution of macromer P3HT-(CH_2_)_3_-VIM and brush polymer P[P3HT-(CH_2_)_3_-VIM] in CHCl_3_/MeOH (*v*/*v* = 1/2), both featuring a single peak with a narrowly dispersed, unimodal particle size distribution. The average hydrodynamic radius (*R*_h_) of the brush polymer P[P3HT-(CH_2_)_3_-VIM] was measured to be ~1130 nm (Figure 7b), which is about one order of magnitude larger than that of the macromer P3HT-(CH_2_)_3_-VIM (Figure 7a), indicating again successful polymerization of the macromer to its corresponding brush polymer.

### 2.3. Synthesis of Linked Donor Polymer-C_60_ Adduct Dyads

With macromers P3HT-(CH_2_)_3_-MIM and P3HT-(CH_2_)_3_-VIM in hand, we envisioned that they could be conveniently converted into linked P3HT-C_60_ donor-acceptor dyads (Scheme 2), an interesting topic of current investigations into photoinduced intramolecular energy transfer and electron transfer within such systems [32,33,34,35,36,47]. The proposed two-step procedure to construct such dyads, P3HT-(CH_2_)_3_-MIM-C_60_ and P3HT-(CH_2_)_3_-VIM-C_60_, first involves deprotonation of the imidazolium chain end to generate the corresponding NHC moiety, followed by facile adduct formation between the polymeric NHC LB and all carbon LA C_60_, a method we established previously for the construction of poly(NHC-C_60_) [41]. Likewise, applying the same procedure to brush polymer P[P3HT-(CH_2_)_3_-VIM] could lead to the novel brush polymer-C_60_ architecture, P[P3HT-(CH_2_)_3_-VIM-C_60_] (Scheme 2c).

**Figure 8:**
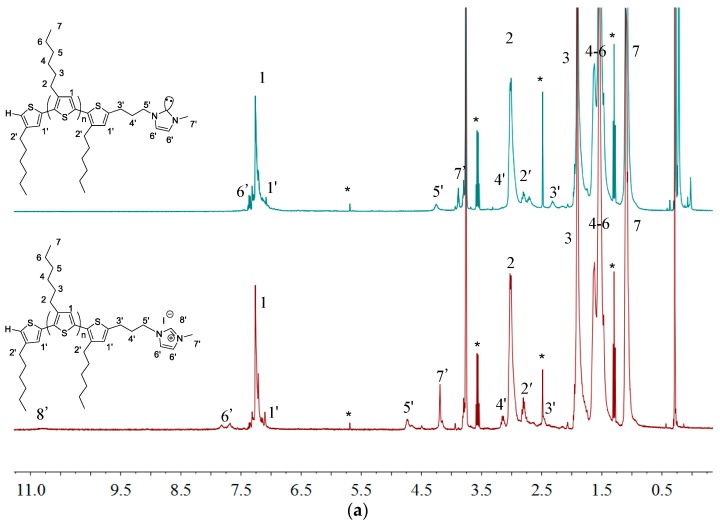

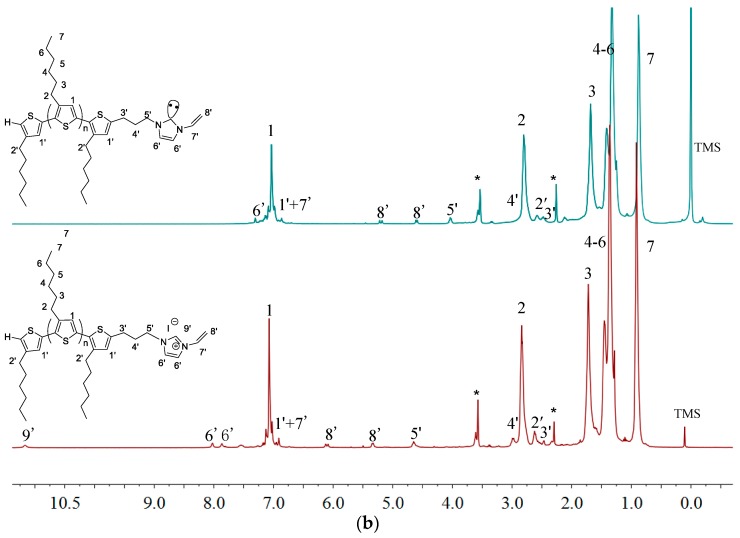
Overlay of ^1^H-NMR (THF-*d*_8_) spectra of P3HT-(CH_2_)_3_-MIM (**a**) and P3HT-(CH_2_)_3_-VIM (**b**) before and after deprotonation. Peaks marked * correspond to residual NMR solvent signals.

**Figure 9:**
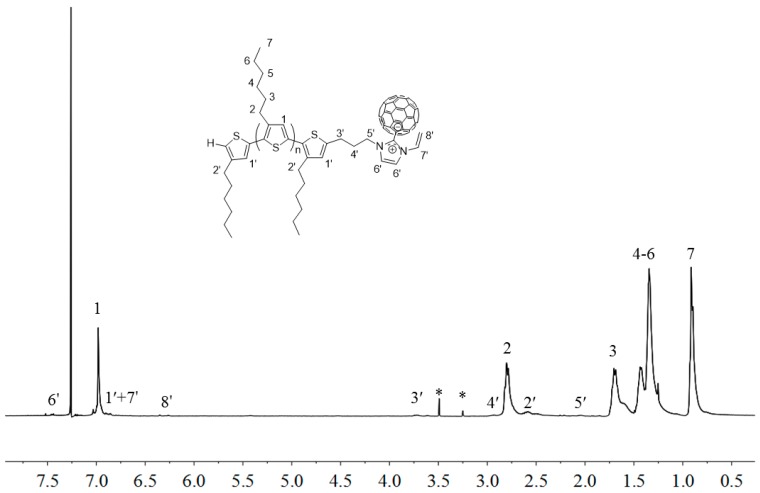
^1^H-NMR (400 MHz, CDCl_3_) spectrum of P3HT-(CH_2_)_3_-VIM-C_60_. Peaks marked * correspond to residual solvent signals. The polymer is only marginally soluble in this NMR solvent.

**Figure 10:**
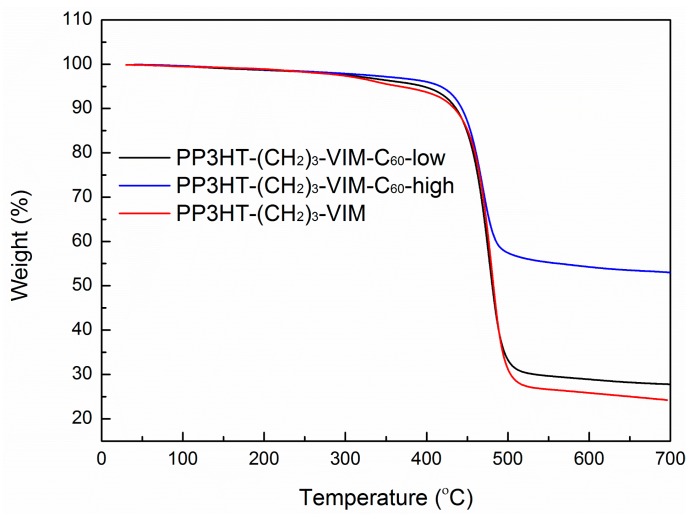
TGA curves of PP3HT-(CH_2_)_3_-VIM, P[P3HT-(CH_2_)_3_-VIM-C_60_-low] and P[P3HT-(CH_2_)_3_-VIM-C_60_-high].

Figure 8 shows overlay ^1^H-NMR spectra P3HT-(CH_2_)_3_-MIM and P3HT-(CH_2_)_3_-VIM before and after the deprotonation reaction, highlighting the disappearance of the acidic methine proton peak of the imidazolium moiety at δ 10.8 ppm (a) and 11.1 ppm (b) upon their respective deprotonation and conversion into the corresponding NHC intermediate. This in situ generated polymeric NHC solution in THF was subsequently treated with C_60_ dissolved in *o*-dichlorobenzene (*o*-DCB) to form the corresponding donor-acceptor dyads as stable adducts P3HT-(CH_2_)_3_-MIM-C_60_ and P3HT-(CH_2_)_3_-VIM-C_60_, accompanied by instantaneous color change to dark orange upon mixing the two solutions. Employing the same two-step procedure but with varied loadings of C_60_ with respect to the brush polymer, two brush polymers of P3HT-C_60_ dyads P[P3HT-(CH_2_)_3_-VIM-C_60_] were conveniently synthesized, either with a low C_60_ loading of P[P3HT-(CH_2_)_3_-VIM]/C_60_ (mol/mol) = 10 (3HT unit)/1 or a high C_60_ loading of P[P3HT-(CH_2_)_3_-VIM]/C_60_ (mol/mol) = 1 (3HT unit)/10), Scheme 2c, Figure 9. The absolute MW of P[P3HT-(CH_2_)_3_-VIM-C_60_] was measured by the GPC-MALS system, with *M*_w_ = 6.70 × 10^4^ g·mol^−1^, *M*_n_ = 4.61 × 10^4^ g·mol^−1^, *Đ* = 1.45. Thermal gravimetric analyzer (TGA) analysis of the two adducts with low and high C_60_ loadings showed a one-step degradation profile (Figure 10). After subtraction of the residue at 700 °C due to the brush polymer itself, C_60_ incorporation can be calculated; for example, for the brush polymer of P3HT-C_60_ dyads prepared with the high C_60_ loading, on average, about 20 P3HT side chains incorporated one C_60_ molecule. It is clear that, due to steric reasons, only a few isolated NHC-carrying side chains can attach a large C_60_ acceptor molecule, thus leaving many reactive NHC sites still unreacted, which were quenched with HBr/MeOH after the adduct formation reaction to regenerate back to the imidazolium moiety, Scheme 2c.

**Figure 11:**
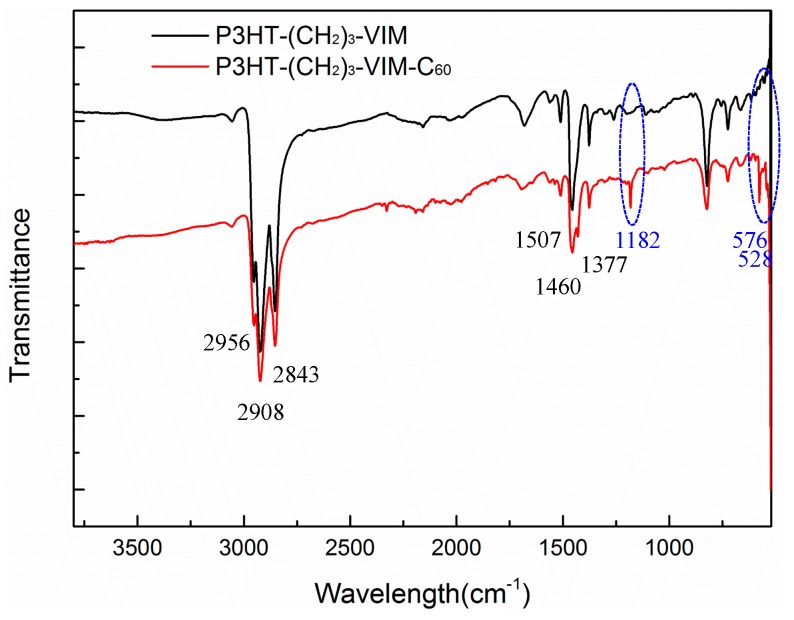
Overlay of FT-IR spectra of P3HT-(CH_2_)_3_-VIM and P3HT-(CH_2_)_3_-VIM-C_60_.

FT-IR spectra of P3HT-(CH_2_)_3_-VIM and its C_60_ dyad, P3HT-(CH_2_)_3_-VIM-C_60_, are compared in Figure 11. As can be seen from the figure, the characteristic bands of P3HT-(CH_2_)_3_-VIM were observed at 2956–2843 cm^−1^ (aliphatic C-H stretch), 1507–1460 cm^−1^ (ring stretch), and 1370–1377 cm^−1^ (methyl deformation) for both P3HT-(CH_2_)_3_-VIM and P3HT-(CH_2_)_3_-VIM-C_60_. However, in the case of the dyad there exhibited additional sharp bands at 1182, 576 cm^−1^ and 528 cm^−1^, characteristic of C_60_, thus confirming that C_60_ (not removable by washing) is covalently linked to the NHC junction connecting the main chain and the brush P3HT side chain via the above described facile LB→LA adduct formation. As expected, macromer P3HT-(CH_2_)_3_-MIM and its C_60_ adduct P3HT-(CH_2_)_3_-MIM-C_60_, as well as brush polymer P[P3HT-(CH_2_)_3_-VIM] and its C_60_ adduct P[P3HT-(CH_2_)_3_-VIM-C_60_] showed same IR spectral features and changes before and after the adduct formation.

### 2.4. Electronic Absorption and Emission Properties

**Figure 12:**
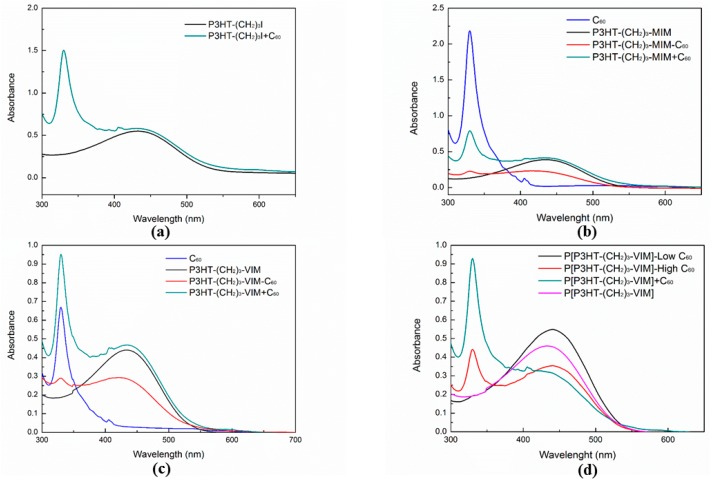
UV-Vis absorption spectra (all in CHCl_3_, 9.4 × 10^−7^ M) of P3HT-(CH_2_)_3_-I and control P3HT-(CH_2_)_3_-I + C_60_ (**a**); P3HT-(CH_2_)_3_-MIM, P3HT-(CH_2_)_3_-MIM-C_60_, and controls C_60_ and P3HT-(CH_2_)_3_-MIM + C_60_ (**b**); P3HT-(CH_2_)_3_-VIM, P3HT-(CH_2_)_3_-VIM-C_60_, and controls C_60_ and P3HT-(CH_2_)_3_-VIM + C_60_ (**c**); P[P3HT-(CH_2_)_3_-VIM], P[P3HT-(CH_2_)_3_-VIM-C_60_-low], P[P3HT-(CH_2_)_3_-VIM-C_60_-high], and control P[P3HT-(CH_2_)_3_-VIM] + C_60_ (**d**).

**Figure 13:**
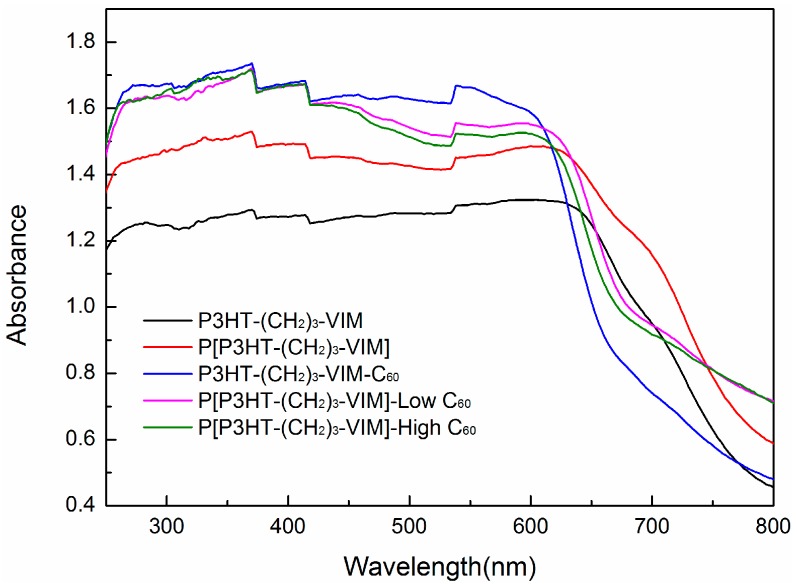
Solid state UV-Vis spectra of P3HT-(CH_2_)_3_-VIM, P[P3HT-(CH_2_)_3_-VIM], P3HT-(CH_2_)_3_-VIM-C_60_, P[P3HT-(CH_2_)_3_-VIM-C_60_-low], and P[P3HT-(CH_2_)_3_-VIM-C_60_-high].

Figure 12 shows the UV-Vis absorption spectra of P3HT-(CH_2_)_3_-I, macromers P3HT-(CH_2_)_3_-MIM and P3HT-(CH_2_)_3_-VIM, brush polymer P[P3HT-(CH_2_)_3_-VIM], as well as their C_60_ adducts and various controls. P3HT-(CH_2_)_3_-I exhibited a single broad absorption band at 432 nm corresponding to the π-π* transition of the 3-hexylthiophene unit. Loading of free C_60_ in the P3HT-(CH_2_)_3_-I solution exerted no effect on the absorption spectrum, besides bringing in characteristic peaks of C_60_ at 406 nm and 330 nm (Figure 12a). As shown in Figure 12b,c, both linked donor-acceptor dyads P3HT-(CH_2_)_3_-MIM-C_60_ (red line) and P3HT-(CH_2_)_3_-VIM-C_60_ (red line) exhibited the combined absorption features of C_60_ and P3HT components, displaying a small shoulder peak at 330 nm and a broad peak at ~437 nm or 434 nm. The linked C_60_ blue-shifted the P3HT absorption maximum by ~22 nm and reduced the molar absorptivity of the P3HT, presumably due to the steric hindrance of the bulky C_60_ that reduces the coplanarity of 3-hexylthiophene units and, thus, the degree of delocalization of π-electrons across the rings. In Figure 12d, brush polymer P[P3HT-(CH_2_)_3_-VIM] (purple line) showed a broad absorption band at 428 nm, as expected for the 3-hexyl-thiophene unit. This band was red-shifted by 6 nm comparing with its macromer P3HT-(CH_2_)_3_-VIM, in agreement with the presence of π-π stacking between the P3HT side chains. Both P[P3HT-(CH_2_)_3_-VIM-C_60_-low] (black line) and P[P3HT-(CH_2_)_3_-VIM-C_60_-high] (red line) possessed absorption features of P3HT at 441 nm, and for the sample with higher C_60_ incorporation showed an additional characteristic peak of C_60_ at 330 nm. Furthermore, solid state UV-Vis spectra (Figure 13) of linear macromer P3HT-(CH_2_)_3_-VIM (black line), brush polymer P[P3HT-(CH_2_)_3_-VIM] (red line), and its C_60_ adducts, P3HT-(CH_2_)_3_-VIM-C_60_ (blue line), P[P3HT-(CH_2_)_3_-VIM-C_60_-low] (purple line) and P[P3HT-(CH_2_)_3_-VIM-C_60_-high] (green line), showed very wide absorption of visible light. The attachment of C_60_ to the polymers via adduct formation apparently enhanced the absorbance of the polymers. All the complementary features of the absorption bands of P3HT-C_60_ dyads and brush polymer of the dyad with that of P3HT broaden the light-harvesting wavelength range of P3HT.

**Figure 14:**
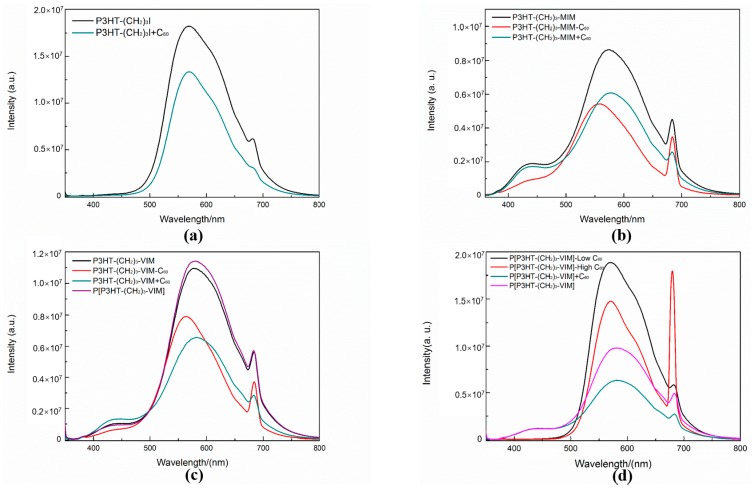
Fluorescence spectra (all in CHCl_3_, 9.4 × 10^−7^ M, excited at the corresponding absorption maxima 342 nm) of P3HT-(CH_2_)_3_-I and control P3HT-(CH_2_)_3_-I + C_60_ (**a**); P3HT-(CH_2_)_3_-MIM, P3HT-(CH_2_)_3_-MIM-C_60_, and control P3HT-(CH_2_)_3_-MIM + C_60_ (**b**); P3HT-(CH_2_)_3_-VIM, P3HT-(CH_2_)_3_-VIM-C_60_, P[P3HT-(CH_2_)_3_-VIM], and control P3HT-(CH_2_)_3_-VIM + C_60_ (**c**); P[P3HT-(CH_2_)_3_-VIM], P[P3HT-(CH_2_)_3_-VIM-C_60_-low], P[P3HT-(CH_2_)_3_-VIM-C_60_-high], and control P[P3HT-(CH_2_)_3_-VIM] + C_60_ (**d**).

The emission properties of the dyads and brush polymer were examined by fluorescence spectroscopy. As shown in Figure 14a, P3HT-(CH_2_)_3_-I displayed a broad fluorescence spectrum with a peak maximum at 569 nm and a small shoulder at 682 nm. Addition of free C_60_ to the solution of P3HT-(CH_2_)_3_-I decreased the emission intensity, because of the fact that the LUMO of C_60_ is lower-lying than that of P3HT-(CH_2_)_3_-I, thus providing a separate route to quench the excited electrons of P3HT-(CH_2_)_3_-I. After modifying the end group of P3HT-(CH_2_)_3_-I by MIM and VIM, a new peak appeared at 443 nm (Figure 14b,c) attributed to the installed IM ring. Interestingly, the linked dyad P3HT-(CH_2_)_3_-MIM-C_60_ solution exhibited a weaker fluorescence intensity than the unlinked mixture of P3HT-(CH_2_)_3_-MIM + C_60_, which indicated an efficient energy/electron transfer from the 3-hexyl-thiophene unit to the C_60_ cage in the photoexcited state, thanks to the covalent linkage between P3HT-(CH_2_)_3_-MIM and C_60_ that shortens the physical distance between the two sites, thereby enhancing the transfer efficiency. Comparing fluorescence spectra of the brush polymer and its C_60_ adducts (Figure 12d), revealed that, upon adduct formation, the characteristic peak of IM disappeared, but the peak at 682 nm became stronger with the increasing loading of C_60_. These observations suggest that the excitons generated in P3HT may contribute to the photocurrent of the solar cells based on such dyads and brush polymer of dyads.

### 2.5. Thermal Behaviors of Macromer, Brush Polymer, and Their C_60_ Adducts

**Figure 15:**
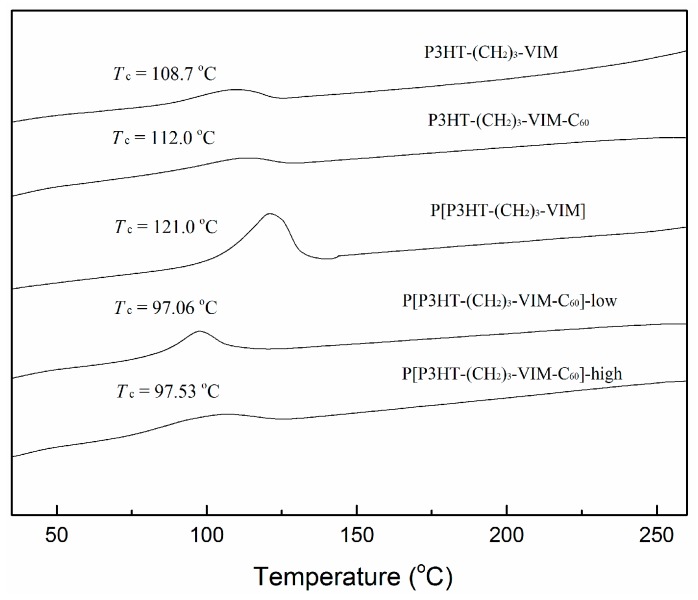
DSC curves showing crystallization temperatures of macromer P3HT-(CH_2_)_3_-VIM, brush polymer P[P3HT-(CH_2_)_3_-VIM], and their C_60_ adducts.

**Figure 16:**
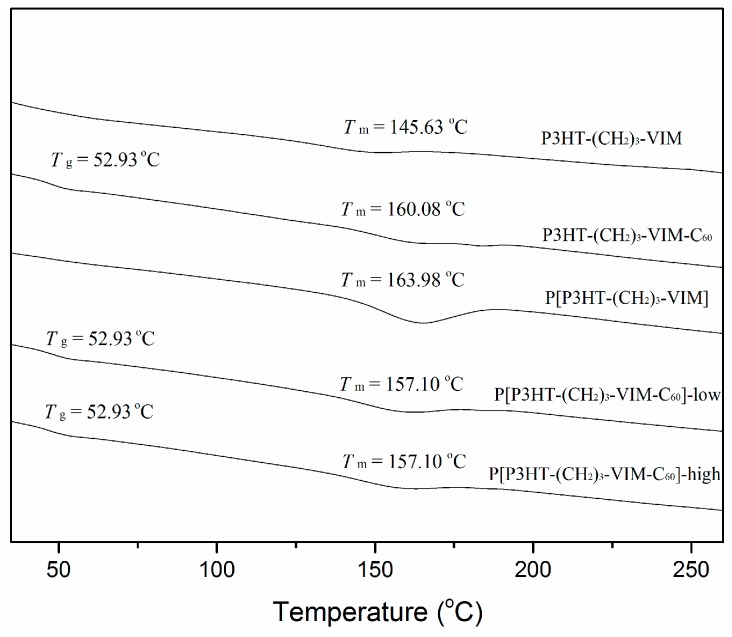
DSC curves showing *T*_g_ and *T*_m_ of macromer P3HT-(CH_2_)_3_-VIM, brush polymer P[P3HT-(CH_2_)_3_-VIM], and their C_60_ adducts.

**Figure 17:**
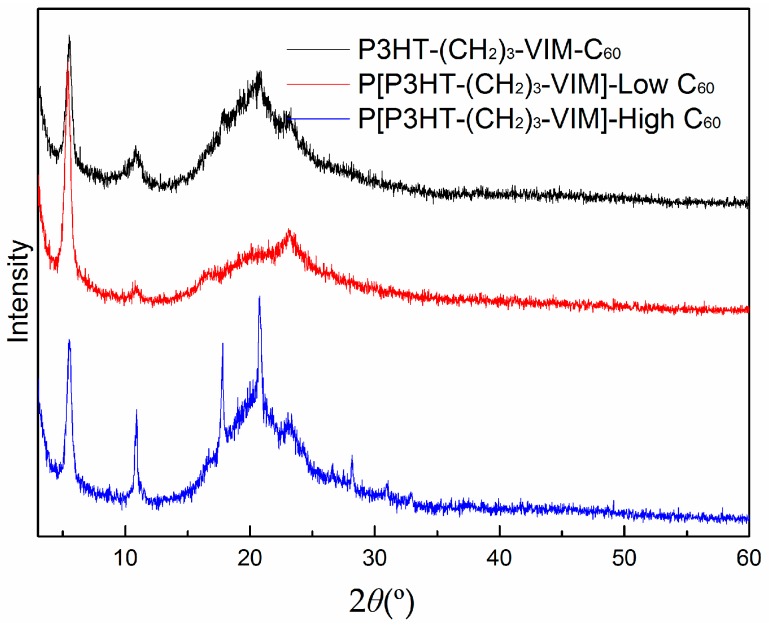
XRD patterns of P3HT-(CH_2_)_3_-VIM-C_60_ and P[P3HT-(CH_2_)_3_-VIM-C_60_].

It is evident that the thermal stability of the polymer and its C_60_ adducts, as defined by *T*_d_ (onset degradation temperature of 10% weight loss in TGA curves) of ~430 °C was kept nearly unchanged with incorporation of C_60_ and increasing of the C_60_ content in the polymer (Figure 10). As expected, however, the stable residue or char yield at 700 °C increased with the amount of C_60_ incorporated. The thermal transition temperatures of macromer P3HT-(CH_2_)_3_-VIM, brush polymer P[P3HT-(CH_2_)_3_-VIM], and their C_60_ adducts were measured by differential scanning calorimetry (DSC) analysis. Typical cooling scan and second heating scans of DSC curves are shown in Figure 15 and Figure 16, displaying crystallization temperature (*T*_c_, Figure 15) as well as glass-transition temperature (*T*_g_) and melting-transition temperature (*T*_m_, Figure 16). The linear macromer P3HT-(CH_2_)_3_-VIM showed a *T*_c_ of 108.7 °C and a *T*_m_ of 145.6 °C, while the brush polymer P[P3HT-(CH_2_)_3_-VIM] exhibited considerably higher thermal transition temperatures with *T*_c_ = 121.0 °C and *T*_m_ = 164.0 °C. In comparison, the thermal properties of linked C_60_ adduct dyads deviated from the polymers, with *T*_c_ = 112.0 °C and *T*_m_ = 160.1 °C for linear polymer adduct P3HT-(CH_2_)_3_-VIM-C_60_ and *T*_c_~97 °C and *T*_m_~157 °C for brush polymer adduct P[P3HT-(CH_2_)_3_-VIM-C_60_]. Overall, according to the above DSC data, the attachment of the bulky C_60_ either as the end group in the case of dyads, or linked to the main chain/side chain junction point in the case of brush polymer, appeared to have some minor impact on the crystallization behavior of the P3HT units. Moreover, all XRD profiles (Figure 17) of P3HT-(CH_2_)_3_-VIM-C_60_ and brush polymer adduct P[P3HT-(CH_2_)_3_-VIM-C_60_] showed a sharp peak at 5.30° to 5.53°, which is associated with the (100) reflection of P3HT [48,49,50,51]. In addition, intense peaks at 10.7° to 10.9°, as well as those appeared at 20.66° and 23.1°, associated with the adduct formation, were also observed. Interestingly, the brush polymer with a high C_60_ loading exhibited sharper peaks at 10.8°, 17.7° and 20.7°, indicating that the higher loading of C_60_ in the adduct enhanced the crystallinity of the brush polymer; this enhancement should facilitate the hole transport to the electrode.

## 3. Experimental Section

### 3.1. Materials, Reagents, and Methods

All synthesis and manipulations with air- and moisture-sensitive chemicals and reagents were performed using standard Schlenk techniques on a dual-manifold Schlenk line or in an inert gas (Ar or N_2_)-filled glovebox. HPLC-grade organic solvents were first saturated with nitrogen during filling of the 20 L solvent reservoirs and then dried by passage through activated alumina (for THF) followed by passage through Q-5 supported copper catalyst (for toluene and hexanes) stainless steel columns. Chloroform-*d*_1_ was dried with 4 Å sieves before used, and THF-*d*_8_ was dried over sodium/potassium alloy and filtered before use.

Reagents 2,5-dibromo-3-hexylthiophene, *t*-BuMgCl, 1,2-bis(diphenylphosphino) ethane nickel(II) chloride [Ni(dppp)Cl_2_], NaI, potassium bis(trimethylsilyl)amide (KHMDS), and *n*-BuLi (1.6 M in THF) were purchased from Sigma-Aldrich Co (St. Louis, MO, USA) and used as received. 1,3-Chlorobromopropane, vinylimidazole and methylimidazole were purchased from TCI America (Portland, OR, USA), distilled and stored in the glovebox for further use. Sodium sulfite (Na_2_SO_3_), *tert*-butyl peroxide (70% solution in water), 2,2′-azobisisobutyronitrile (AIBN), dibenzoyl peroxide (BPO), and 2,2-dimethoxy-2-phenylacetophenone (DMPA) were purchased from TCI America and used as received. C_60_ was purchased from Sigma-Aldrich and dried under vacuum at 60 °C before use.

Polymer number-average molecular weight (*M*_n_) and molecular weight distribution or dispersity (*Đ* = *M*_w_/*M*_n_) were measured by gel permeation chromatography (GPC) analyses carried out at 40 °C and at a flow rate of 1.0 mL min^−1^, with CHCl_3_ as an eluent, on a Waters 1515 GPC instrument (Waters Corporation, Milford, MA, USA) equipped with one PLgel 5 μm guard and three PLgel 5 μm mixed-C columns (Polymer Laboratories, Waters Corporation, Milford, MA, USA ; linear range of molecular weight = 200–2,000,000 g/mol). The instrument was calibrated with polystyrene standards, and chromatograms were processed with Waters Empower software (version 2002, Waters Corporation, Milford, MA, USA). Polymer absolute weight-average molar weight (*M*_w_) and dispersity *Đ* were determined by GPC-MALS coupled with a multi-angle light scattering detector (DAWN HELEOS 8, Wyatt Technology Corporation, Santa Barbara, CA, USA)) in THF (1.0 mL/min) at 35 °C. Crystallization temperatures (*T*_c_), glass transition temperatures (*T*_g_), and melting-transition temperatures (*T*_m_) of the polymers were measured by differential scanning calorimetry (DSC) on a Q20 DSC, TA Instruments. Samples were first heated until 280 °C at 10 °C min^−1^, cooled to 25 °C at 10 °C min^−1^, and then reheated again at 10 °C min^−1^ to 250 °C. All *T*_g_ and *T*_m_ values were obtained from the second heating scan, after removing the thermal history. Thermal properties of the polymers were also analyzed by thermal gravimetric analyzer (TGA), on a Q50 TGA (TA Instruments, New Castle, DE, USA); polymer samples were heated from ambient temperature to 700 °C at a heating rate of 10 °C/min under a nitrogen atmosphere. Selected macromer samples were analyzed by matrix-assisted laser desorption/ionization time-of-flight mass spectroscopy (MALDI-TOF MS); the experiment was performed on a Ultraflex MALDI-TOF mass spectrometer (Bruker Daltonics, Billerica, MA, USA) operated in positive ion, reflector mode using a Nd: YAG laser at 355 nm and 25 kV accelerating voltage. External calibration was done using a peptide calibration mixture (4–6 peptides) on a spot adjacent to the sample. The raw data were processed in the FlexAnalysis software (version 2.4, Bruker Daltonics).

NMR spectra were recorded on an Inova 400 MHz spectrometer (Varian, Palo Alto, CA, USA) and chemical shifts were referenced to residual undeuterated solvent resonances and are reported as parts per million relative to SiMe_4_. Fourier transform infrared (FTIR) spectroscopy was performed on a Nicolet iS50 FT-IR spectrometer (Thermo Fisher Scientific, Waltham, MA, USA) equipped with a diamond attenuated totalreflectance at room temperature in the range of 550–4000 cm^−1^. UV-Vis measurements were recorded on a Cary UV-Vis-NIR spectrometer (Agilent, Santa Clara, CA, USA) equipped with a variable angle specular reflectance accessory. Fluorescence spectra were measured using a Fluorolog-TAU-3 spectrofluorimeter (Jobin-Yvon, Edison, NJ, USA). Dynamic light scattering (DLS) was used to determine the average hydrodynamic radii (*R*_h_) of polymer samples. X-ray diffraction analysis was carried out by an X-ray diffractometer (D8 Advance, Bruker, Billerica, MA, USA) with a step time of 188.4 s and a step size of 0.001°. The radiation was a monochromatized Cu*Kα* beam with a wavelength of *k* = 1.54060 Å. The device created X-ray via an 18 kW rotating copper anode X-ray tube. The X-ray was then collimated with user-defined slits, monochromated, and targeted on the sample. Reflected interference patterns from the sample were then recorded. The DLS analyses of the dilute polymer solutions (typically at 7.3 × 10^−4^ g/mL unless indicated otherwise) were performed on a DynaPro Titan (Wyatt Technology Corporation, Santa Barbara, CA, USA, model 803) equipped with a temperature controller and a 25 mW laser operating at λ = 826.6 nm, using Dynamics (Version 6, Wyatt Technology Corporation, Santa Barbara, CA, USA) data collection and analysis software. The samples were gently dissolved in chloroform and then diluted with methanol. The DLS measurements were conducted at 25 °C with a scattering angle of 90°, and the histograms were obtained by the averaging of ten runs, with each run of 10 s in duration.

### 3.2. Preparation of P3HT with H/H Chain-Ends, H-P3HT-H

Literature procedures [42] were modified to prepare the H/H end-capped P3HT. 2,5-Dibromo-3-hexylthiophene (1.5 g, 4.6 mmol), *t*-BuMgCl (5.06 mmol) and THF (9 mL) were added to a 100 mL Schlenk flask, and the mixture was stirred at room temperature for 20 h. The obtained yellow solution was transferred to a 100 mL Schlenk flask which contained Ni(dppp)Cl_2_ (40.5 mg in 20 mL THF) using cannula, and the resulting mixture was stirred for 12 min. The polymerization was stopped by addition of 5 mL of 5.0 M HCl. After precipitation in methanol, the polymer was filtered into a Soxhlet thimble and extracted with methanol and hexanes overnight to wash away the residual monomer and low molecular weight impurities. The purified polymer was obtained in 70% yield after redissolution in CHCl_3_, precipitation in methanol, and drying. ^1^H-NMR (CDCl_3_), (δ): 6.98 (s), 6.70 (s, 3-hexyl-thiophene chain end), 2.80 (t), 2.59 (m, 3-hexylthiophene chain end), 1.70 (m), 1.44 (m), 1.36 (m), 0.92 (t) ppm. The ^1^H-NMR spectrum of H-P3HT-H is shown in Figure 1. Except for the characteristic peaks of the P3HT main chain, the chain ends can also be observed at 6.70 and 2.60 ppm, attributed to the proton of the chain end’s double bond and the protons adjacent to the thiophene ring. The molecular weight (MW) can be calculated according to the following formula: MW(NMR) = (2I_2.80_ ppm/I_2.60_ ppm) × 166 + 167 + 167. Accordingly, the MW of H-P3HT-H was calculated to be ~2000 g/mol for the sample prepared by using the monomer/Ni ratio of 20/1.

### 3.3. Synthesis of P3HT-(CH_2_)_3_I

H-P3HT-H (1.0 g, 0.5 mmol) was dried overnight and dissolved in 80 mL of THF under nitrogen in a Schlenk flask. The solution was cooled to −30 °C and *n*-BuLi (0.17 mL, 0.275 mmol) was added dropwise under nitrogen. The reaction was allowed to stir at 0 °C for 2 h. Then, 1-bromo-3-chloropropane (0.027 mL, 0.275 mmol) was added dropwise into the solution. The reaction was allowed to warm to room temperature overnight. The resulting reaction mixture was poured into cold methanol to precipitate the macromer product, which was isolated by filtration and drying under vacuum; yield: 0.95 g, 95%. The obtained P3HT-(CH_2_)_3_Cl (0.95 g, 0.475 mmol) and NaI (0.71 g, 4.75 mmol) were dissolved into the mixed solvent of 50 mL THF and 30 mL acetone and refluxed for 24 h. The reaction mixture was poured into cold methanol to precipitate the polymer and washed with water several times to remove the salt. The yield of the polymer was 0.80 g (80.8%), obtained by redissolution in CHCl_3_ and precipitation in methanol. ^1^H-NMR (CDCl_3_), δ): 6.98 (s), 6.86 (s, 3-hexyl-thiophene chain end), 3.27 (t, -CH_2_CH_2_CH_2_I chain end), 2.88 (t, -CH_2_CH_2_CH_2_I chain end), 2.80 (t), 2.62, 2.57 (m, 3-hexyl-thiophene chain end), 2.17 (t, -CH_2_CH_2_CH_2_I chain end), 1.71 (m), 1.44 (m), 1.35 (m), 0.91 (t) ppm. ^13^C-NMR (CDCl_3_), δ): 142.50, 133.87, 130.45, 128.69, 31.58, 30.49, 29.24, 22.78, 14.11.

### 3.4. Synthesis of P3HT-(CH_2_)_3_-MIM

A mixture of P3HT-(CH_2_)_3_I (0.80 g, 0.4 mmol) and 1-methylimidazole (49.3 mg, 0.6 mmol) in THF (50 mL) was refluxed for 6 days in a nitrogen atmosphere. After evaporation of most of the solvent, the mixture was poured into cold methanol to precipitate the polymer. The yield of the polymer was 0.70 g (84.3%), obtained by redissolution in CHCl_3_ and precipitation in methanol. ^1^H-NMR (CDCl_3_), δ): 10.62 (s, -CH- at MIM end), 7.19 (s, -*CH=CH*- at MIM end), 6.98 (s), 6.87 (s, 3-hexyl-thiophene chain end), 4.43 (t, -CH_2_CH_2_CH_2_- chain end), 4.10 (s, -CH_3_ at MIM end), 2.96 (t, -CH_2_CH_2_CH_2_- chain end), 2.78 (t), 2.59, 2.50 (m, 3-hexyl-thiophene chain end), 2.34 (t, -CH_2_CH_2_CH_2_- chain end), 1.68 (m), 1.42 (m), 1.33 (m), 0.91 (t) ppm. ^13^C-NMR (CDCl_3_), δ): 139.86, 133.66, 128.56, 31.67, 30.49, 29.44, 29.24, 14.11.

### 3.5. Synthesis of P3HT-(CH_2_)_3_-VIM

A mixture of P3HT-(CH_2_)_3_I (0.80 g, 0.4 mmol) and 1-vinylimidazole (56.5 mg, 0.6 mmol) in THF (50 mL) was refluxed for 6 days in a nitrogen atmosphere. After evaporation of most of the solvent, the mixture was poured into cold methanol to precipitate the polymer. The yield of the polymer was 0.75 g (87.6%), obtained by redissolution in CHCl_3_ and precipitation in methanol. ^1^H-NMR (CDCl_3_), δ): 11.22 (s, -CH- at VIM end), 7.42 (s, -*CH=CH*- at VIM end), 6.98 (s), 6.86 (s, 3-hexyl-thiophene chain end and -CH=CH_2_ at VIM end), 5.92 and 5.48 (m, -CH=CH_2_ at VIM end), 4.54 (t, -CH_2_CH_2_CH_2_- chain end), 2.94 (t, -CH_2_CH_2_CH_2_- chain end), 2.80 (t), 2.51 (m, 3-hexylthiophene chain end), 2.38 (t, -CH_2_CH_2_CH_2_- chain end), 1.70 (m), 1.42 (m), 1.34 (m), 0.91 (t) ppm. ^13^C-NMR (CDCl_3_), δ): 139.86, 133.66, 130.45, 128.56, 31.68, 30.49, 29.45, 22.64, 14.11.

### 3.6. Polymerization of P3HT-(CH_2_)_3_-VIM

Radical polymerization of the macromer was performed in a 20 mL, oven-dried Schlenk reactor inside an oil bath. In a typical procedure, *tert*-butyl hydroperoxide was premixed with 0.5 equimolar amount of Na_2_SO_3_ in THF for ca. 5 min. The polymerization was started by addition of a macromer P3HT-(CH_2_)_3_-VIM solution in THF. After the prescribed time, the polymerization was immediately quenched by pouring the reaction solution into 5% HCl-acidified methanol. The polymer was collected by filtering, washing several times with methanol, and drying in a vacuum oven at 50 °C to a constant weight.

### 3.7. Synthesis of Polymer-C_60_ Adducts: P3HT-(CH_2_)_3_-MIM-C_60_, P3HT-(CH_2_)_3_-VIM-C_60_ and P[P3HT-(CH_2_)_3_-VIM-C_60_]

KHMDS (0.0253 g, 0.13 mmol) (15% *w*/*w* in toluene) was added dropwise into a solution of P3HT-MIM (0.1535 g, 0.11 mmol) in 3 mL THF, during which time the color changed from orange to dark orange. C_60_ (0.087 g, 0.12 mmol) was dissolved in 2.0 mL *o*-DCB and added into the above solution, causing a color change into more intensively dark orange. After being stirred overnight, the solvent was removed and THF was added to the residue to precipitate the excess C_60_ (the solubility of C_60_ in THF is 0.006 mg/mL). The solution was filtered to remove the excess C_60_, and the filtrate was added HBr/MeOH to stabilize the unreacted carbene sites. Next, hexanes were added to wash polymer, which was dried under vacuum. The preparation of P3HT-(CH_2_)_3_-VIM-C_60_ and P[P3HT-(CH_2_)_3_-VIM-C_60_] followed the same procedures as for the preparation of P3HT-(CH_2_)_3_-MIM-C_60_.

## 4. Conclusions

In summary, we have designed and synthesized two P3HT macromers with two different imidazolium end groups, P3HT-(CH_2_)_3_-MIM and P3HT-(CH_2_)_3_-VIM, the latter of which carries a polymerizable vinyl group. Using the combined “graft through” and radical polymerization method, macromer P3HT-(CH_2_)_3_-VIM was successfully polymerized into a novel brush polymer, P[P3HT-(CH_2_)_3_-VIM], comprising a poly(vinyl imidazolium) backbone and P3HT side chains. Utilizing the instantaneous adduct forming reaction between the P3HT-NHC LB and all carbon LA C_60_, two linked donor-acceptor dyads, P3HT-(CH_2_)_3_-MIM-C_60_ and P3HT-(CH_2_)_3_-VIM-C_60_, as well as brush polymer of dyads, P[P3HT-(CH_2_)_3_-VIM-C_60_], have been synthesized by a convenient one-pot, two-step procedure involving first deprotonation of the imidazolium moiety to the corresponding NHC LB, followed by addition of the LA C_60_. Investigations into the thermal behaviors of the resulting polymeric P3HT-C_60_ dyads and brush polymer of dyads revealed that the attachment of the bulky C_60_, either as the end group in the case of dyads or linked to the main chain/side chain junction point in the case of the brush polymer, appeared to have some minor impact on the crystallization behavior of the P3HT units. However, a change of the topology of the P3HT-C_60_ dyad from linear to brush architecture enhanced the crystallinity and *T*_m_ of the P3HT domain. Electronic absorption and emission properties of the resulting polymeric dyads and brush polymer of dyads, when compared with control systems where C_60_ is either absent or present freely (unlinked), indicated that the donor-acceptor adduct formation that covalently links the donor-acceptor domains together provides a promising approach to extend the light-harvesting wavelength range of the active layer and increase the generation efficiency of excitons of the semiconducting polymer. Hence, we anticipate that this novel brush polymer architecture of the linked donor-acceptor dyads could provide some interesting or unique features complementing the currently available architectures of the active layer domains of critical importance to performances of OPV devices.
